# CO_2_ Capture Mechanism by Deep Eutectic Solvents Formed by Choline Prolinate and Ethylene Glycol

**DOI:** 10.3390/molecules28145461

**Published:** 2023-07-17

**Authors:** Mingzhe Chen, Jinming Xu

**Affiliations:** School of Science, China University of Geosciences, Beijing 100083, China; 2019220039@email.cugb.edu.cn

**Keywords:** CO_2_ capture, absorption, deep eutectic solvents, mechanism, proline

## Abstract

The choline prolinate ([Ch][Pro]) as a hydrogen bond acceptor and ethylene glycol (EG) as a hydrogen bond donor are both used to synthesize the deep eutectic solvents (DESs) [Ch][Pro]-EG to capture CO_2_. The CO_2_ capacity of [Ch][Pro]-EG is determined, and the nuclear magnetic resonance (NMR) and infrared (IR) spectrum are used to investigate the CO_2_ capture mechanism. The results indicate that CO_2_ reacts with both the amino group of [Pro]^−^ anion and the hydroxyl group of EG, and the mechanism found in this work is different from that reported in the literature for the [Ch][Pro]-EG DESs.

## 1. Introduction

Carbon dioxide (CO_2_) emissions have increased at an unbelievable rate, which causes great concern in global society and results in increasing atmospheric temperature and rising sea levels. The unprecedented amounts of atmospheric CO_2_ are mainly emitted from the combustion of fossil fuels in industry [1,2,3]. An urgent demand to reduce the rising levels of CO_2_ has driven different industries and fields to explore efficient CO_2_ capture technologies. The current prominent commercial method for CO_2_ capture in the industry is the alkanolamine-based scrubbing process, which mainly utilizes aqueous solutions of alkanolamines to absorb CO_2_ chemically [4,5]. However, alkanolamine-based sorption systems have several inherent drawbacks, such as solvent degradations, equipment corrosion, and high absorbent regeneration energy [6,7]. Thus, developing new efficient sorption systems capable of avoiding the above-mentioned drawbacks is one of the main challenges in the field of carbon capture and storage [8].

In the past decades, as an alternative to amine-based absorbents, ionic liquids (ILs) have received a lot of attention in the CO_2_ capture field because of their attractive properties, including negligible vapor pressure, high thermal and chemical stability, low flammability, and tunable structures [9,10]. To date, a number of functionalized ILs, which can chemically capture CO_2_, have been investigated for CO_2_ capture. Among them, anion-functionalized ILs, such as azolide-based and amine-based ILs, exhibit high CO_2_ absorption capacities [11,12,13]. However, the main shortcoming of the functionalized ILs is their high viscosity, which limits their large-scale industrial application.

In recent years, deep eutectic solvents (DESs), emerging as a new kind of solvent, have gained significant attention because they share many features with ILs, such as very low vapor pressure and tunable properties [14,15]. At present, most DESs are formed by combining hydrogen bond donors (HBDs) and hydrogen bond acceptors (HBAs), and the intermolecular hydrogen bonds formed between HBDs and HBAs induce a large depression of melting or freezing temperatures of DESs [16,17]. Due to their attractive properties, DESs have been widely investigated in many fields, including organic synthesis, catalysis, biodiesel conversion, electrochemistry, and nanotechnology [18,19,20,21,22,23].

Moreover, the applications of DESs to capture CO_2_ have also been widely studied [24,25]. Many DESs formed by halide anion-based quaternary ammonium/phosphonium salts with common HBDs (ethylene glycol, glycerol, lactic acid, decanoic acid, acetic acid, urea, etc.) have been utilized to capture CO_2_, and these DESs exhibit low CO_2_ capacities because they physically interact with CO_2_ [26,27,28,29,30,31]. In order to improve the CO_2_ capacity of DESs, several works introduce amino groups into the components (HBDs or HBAs) of DESs. These amino-contained DESs, which are amino-functionalized DESs, present a high capacity due to the chemical interactions between amino groups and CO_2_ [32,33,34,35,36,37]. Zhang et al. reported the anime-functionalized DESs composed of 1-butyl-3-methylimidazolium chloride (BmimCl) and monoethanolamine (MEA) for CO_2_ capture [32], and results indicated that CO_2_ reacted with the amino group of MEA by forming a carbamate species and BmimCl:MEA (1:4, molar ratio) exhibited a high CO_2_ capacity (21.4 wt%) at 25 °C and 100 kPa. Trivedi and co-authors studied CO_2_ capture by DESs formed by monoethanolammonium chloride ([MEAH][Cl]) and ethylenediamine (EDA), and the DESs [MEAH][Cl]-EDA also exhibited a high capacity through the reaction between CO_2_ and EDA. [33] Shukla et al. investigated CO_2_ absorption by DESs composed of ammonium salts-based HBAs (choline chloride (ChCl), tetrabutylammonium bromide (TBAB), etc.) and amine-based HBDs (MEA, EDA, tetraethylenepentamine (TEPA), etc.), finding that the synergistic interactions between HBAs and HBDs were important factors for CO_2_ capture by DESs [34]. Wu and co-authors synthesized DESs comprising triethylenetetrammonium chloride ([TETAH][Cl]) and EG or diethylene glycol (DG), and [TETAH][Cl]:EG(1:3) could absorb CO_2_ up to 17.5 wt% at 1 atm and 40 °C [35]. Then, hydrophobic amine-functionalized DESs were developed for carbon capture. The hydrophobic DESs [TETAH][Cl]: Thymol (1:3) could chemically capture CO_2_ through the reaction between CO_2_ and the free amino groups on the cation [TETAH]^+^ [36].

Functionalized DESs containing basic anions in the solvents are also proposed to capture carbon. It is found that anion-functionalized DESs, consisting of EG and solid azolide ILs (tetraethylphosphonium imidazolide ([P_2222_][Im]), tetraethylphosphonium 1,2,4-triazolide ([P_2222_][Triz]), etc.), could chemically absorb CO_2_ through the reaction between CO_2_ and EG [38]. Interestingly, CO_2_ absorption behaviors of anion-functionalized DESs based on 1,2,3-triazole (Tz) can be changed by tuning the strength of hydrogen bonds formed between the anion [Tz]^−^ and HBDs (EG or Tz) [39]. The DESs composed of 1-ethyl-3-methylimidazolium 2-cyanopyrrolide ([Emim][2-CNpyr]) and EG can also chemically capture CO_2_, and CO_2_ can react with both [Emim][2-CNpyr] and EG [40]. The CO_2_ absorption by phenol-derived anion-functionalized DESs was also studied [41], and the results indicated that the steric hindrance of functional groups of HBDs greatly impacted the absorption mechanisms. For example, when CO_2_ was captured by [Et_4_N][Car]-EG ([Et_4_N][Car]: tetraethylammonium carvacrolate), CO_2_ reacted with both the anion [Car]^−^ and EG. Nevertheless, for the [Et_4_N][Car]-4CH_3_-Im (4CH_3_-Im: 4-methylimidazole) system, CO_2_ reacted with the anion [Car]^−^, but did not react with 4CH_3_-Im. In addition, superbase-derived anion-functionalized DESs were also explored for CO_2_ capture, such as [DBUH][Im]-EG (DBU: 1,8-diazabicyclo-[5,4,0]undec-7-ene) [42], [DBUH][MLU]-EG (MLU: methyl urea) [43], [DBNH][Triz]-EG (DBN: 1,5-Diazabicyclo[4.3.0]non-5-ene) [44], [DBUH][Car]-EG [45], DBN-EU (EU: 2-imidazolidone) [46], [DBUH][4-F-PhO]-EG(4-F-PhO:4-Fluorophenolate) [47], ternary DESs [DBUH][4-F-PhO]-4-F-PhOH-EG [47], and DBN-BmimCl-Im [48]. According to the results reported in the literature, it can be found that the components of DESs greatly affect the CO_2_ absorption behaviors of functionalized DESs. Therefore, revealing the interactions between CO_2_ and the components of DESs is of great importance to the design of efficient DESs [49].

**Scheme 1:**
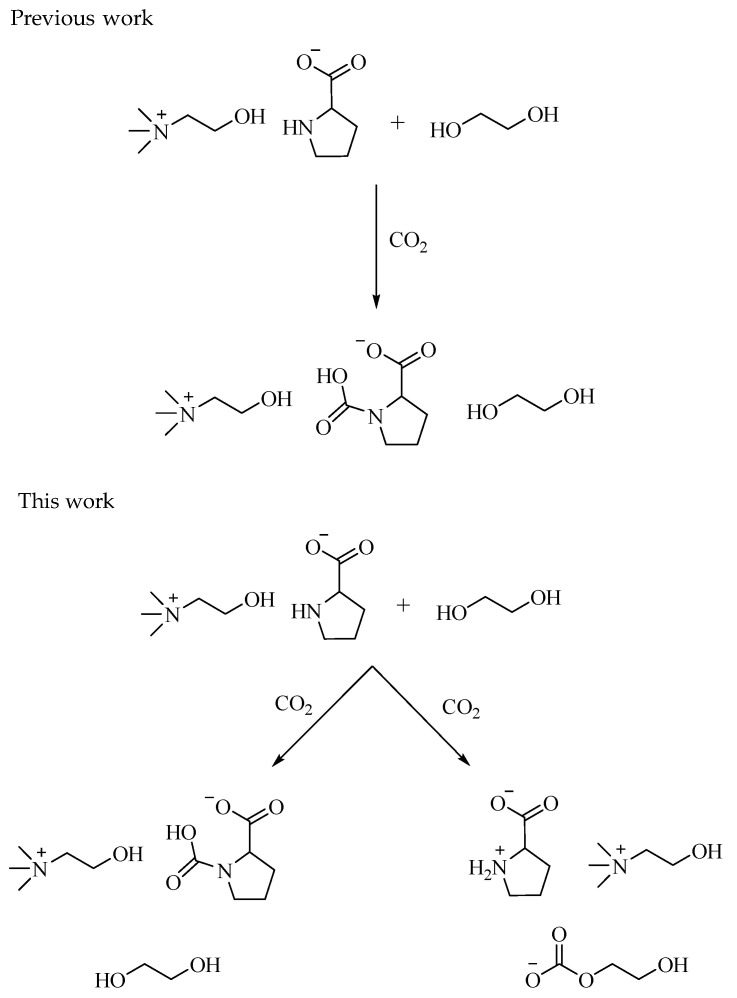
The CO_2_ capture mechanism by [Ch][Pro]-EG reported in previous work [50] and found in this work.

Recently, the amino-functionalized DESs, consisting of choline prolinate ([Ch][Pro]) and EG, were prepared to capture CO_2_, and the mechanism studies suggested that CO_2_ reacted with the anion [Pro]^−^, forming carbamate acid, but not with EG [50] (Scheme 1). In this work, we also investigate the CO_2_ capture mechanism by [Ch][Pro]-EG DESs. However, based on the nuclear magnetic resonance (NMR) and Fourier transform infrared (FTIR) results, we find that CO_2_ can react with both the anion [Pro]^−^ and EG (Scheme 1), and the discussion can be found in the sections below.

## 2. Results and Discussion

**Figure 1:**
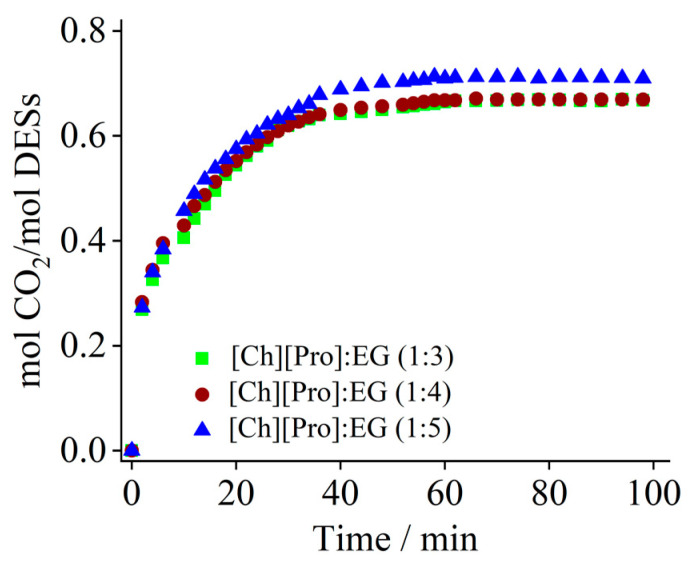
The CO_2_ capture by [Ch][Pro]-EG DESs at 25 °C and 1.0 atm.

The CO_2_ capacities of [Ch][Pro]-EG solvents are determined at 25 °C and 1.0 atm (Figure 1). As seen in Figure 1, [Ch][Pro]:EG (1:3, molar ratio), (1:4), and (1:5) could capture 0.66, 0.67, and 0.71 mol CO_2_/mol DESs, respectively.

**Figure 2:**
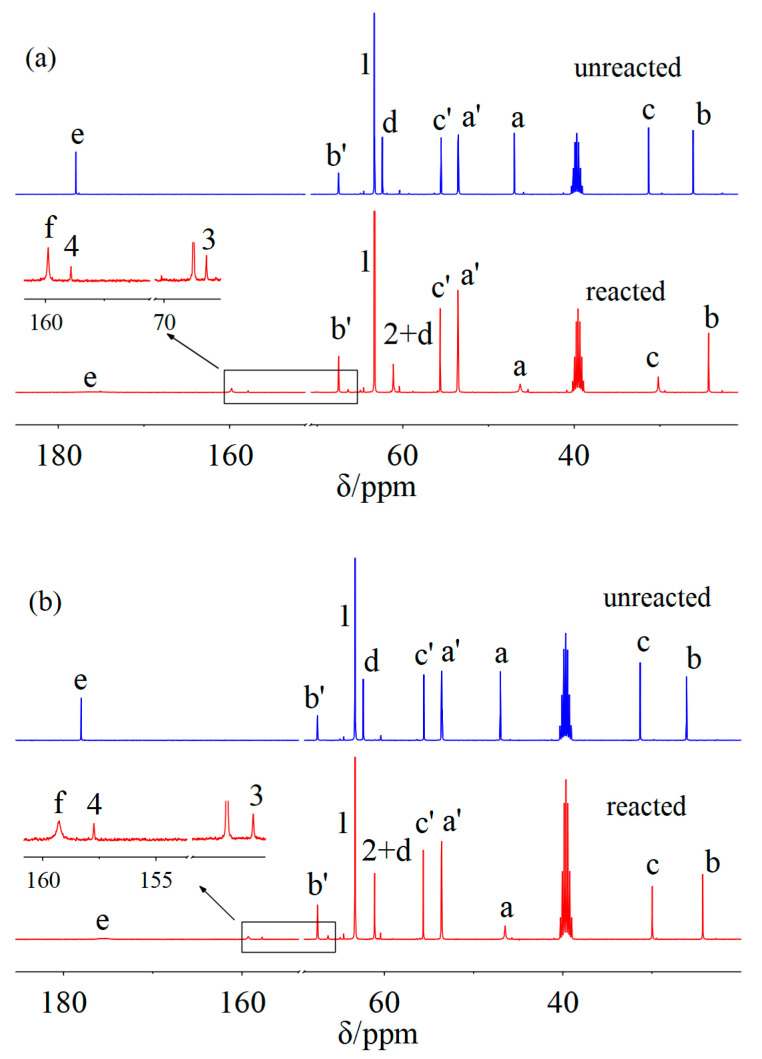
The ^13^C NMR spectra of [Ch][Pro]:EG (1:3) (**a**) and [Ch][Pro]:EG (1:5) (**b**) before and after CO_2_ capture. Letters from a to e are the labels of carbon atoms of the [Pro]^−^ anion with and without CO_2_. Letter f is the carbonyl carbon attached to the N atom of [Pro]^−^ anion. Letters from a’ to c’ are the lables of carbon atoms of the cation [Ch]^+^. Numer 1 is the carbon atom of EG. Numbers 2–4 are the carbon atoms of EG-based carbonates.

Then, NMR and FTIR methods are utilized to study the interactions between CO_2_ and solvents. The ^13^C NMR spectra of [Ch][Pro]:EG (1:3) before and after CO_2_ capture are presented in Figure 2a. After CO_2_ capture, new peaks can be observed at 159.7 (C-f), 157.8 (C-4), and 66.4 (C-3) ppm, and the signal of C-d carbon of the anion [Pro]^−^ shifts upward from 62.2 to 61.1 ppm. The new peak at 159.7 ppm is ascribed to the carbonyl carbon of carbamate acid, suggesting the CO_2_ reacts with the anion [Pro]^−^ [50]. The new peaks at 157.8 ppm can be attributed to the carbonyl carbon of the carbonate species formed by the reaction between CO_2_ and EG [39,42,43]. The peak at 66.4 ppm is the methylene carbon of the carbonate species derived from EG. The other methylene carbon (C-2) peak of carbonate is overlapped with the C-d carbon (61.1 ppm). The NMR results of [Ch][Pro]:EG (1:4) and (1:5) show a similar phenomenon, and the NMR spectra of [Ch][Pro]:EG (1:5) before and after CO_2_ capture are shown in Figure 2b. The new peaks after capture are at 159.2 (C-f), 157.6 (C-4), and 66.2 (C-3) ppm for [Ch][Pro]:EG (1:5), indicating again that CO_2_ reacts with both the anion [Pro]^−^ and EG.

**Figure 3:**
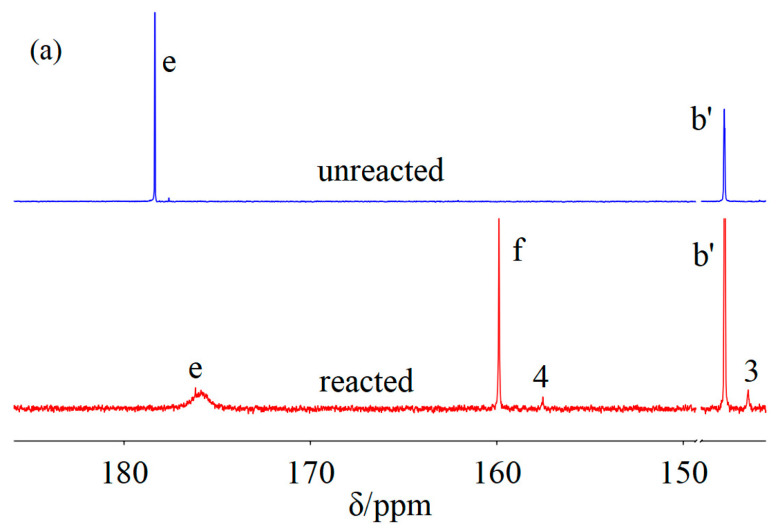

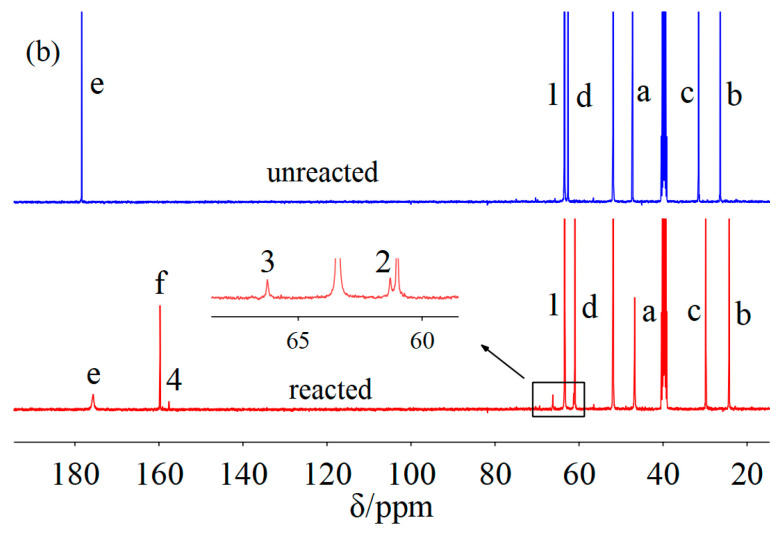
The ^13^C NMR spectra of [Ch][Pro]:EG (1:2) (**a**) and [Et_4_N][Pro]:EG (1:3) (**b**) before and after CO_2_ capture. Letters from a to e are the labels of carbon atoms of the [Pro]^−^ anion with and without CO_2_. Letter f is the carbonyl carbon attached to the N atom of [Pro]^−^ anion. Numer 1 is the carbon atom of EG. Numbers 2–4 are the carbon atoms of EG-based carbonate.

The ^13^C NMR spectra of [Ch][Pro]:EG (1:2) before and after absorption are also analyzed. As presented in Figure 3a, it can be seen that there are new peaks at 159.6 (C-f), 157.3 (C-4), and 66.0 (C-3) ppm after capture, confirming that CO_2_ reacts with EG in the [Ch][Pro]:EG (1:2), which is different from the results reported by Klemm et al. [50]. Furthermore, the ^13^C NMR spectra of [Et_4_N][Pro]:EG (1:3) ([Et_4_N][Pro]: tetraethylammonium prolinate) are also recorded to test whether CO_2_ can react with EG after changing cations of DESs. As shown in Figure 3b, four new peaks can be found at 159.5 (C-f), 157.4 (C-4), 66.0 (C-3), and 61.1 (C-2) ppm after capture, suggesting CO_2_ can also react with both the anion [Pro]^−^ and EG in the [Et_4_N][Pro]:EG (1:3) solvent, which is consistent with the [Ch][Pro]-EG DESs. Fortunately, the C-2 peak ascribed to one of the methylene carbons of EG-based carbonate species can be observed (Figure 3b), and it is not overlapped with C-d carbon this time. The full-window ^13^C NMR spectra of the solvents used in this work are shown in Figures S1–S5.

The ^1^H NMR spectra of the solvents used before and after CO_2_ capture are presented in Figures S6–S10. As can be seen in the ^1^H NMR spectra of the DESs after CO_2_ capture, the methylene hydrogen (H-3) of EG-based carbonate can be observed. Based on the H-3 peak integral values, the amount of CO_2_ captured by EG can be calculated. The loadings of EG-bonded CO_2_ of [Ch][Pro]:EG (1:2), (1:3), (1:4), and (1:5) are 0.07(11.3%), 0.10 (15.2%), 0.13 (19.4%), and 0.14 (19.7%) mol CO_2_/mol DESs, respectively. The values in parenthesis are the percentages of EG-bonded CO_2_ to the overall CO_2_ capacity. The results indicate that EG-bonded CO_2_ rise with increasing the amount of EG in the DESs. The results reported by reference [50] claimed that CO_2_ did not react with EG in the DESs [Ch][Pro]:EG (1:2), probably because the intensities of the H and C peaks of EG-based carbonate in NMR spectra were very weak and these peaks can be easily overlooked if they are not carefully identified.

**Figure 4:**
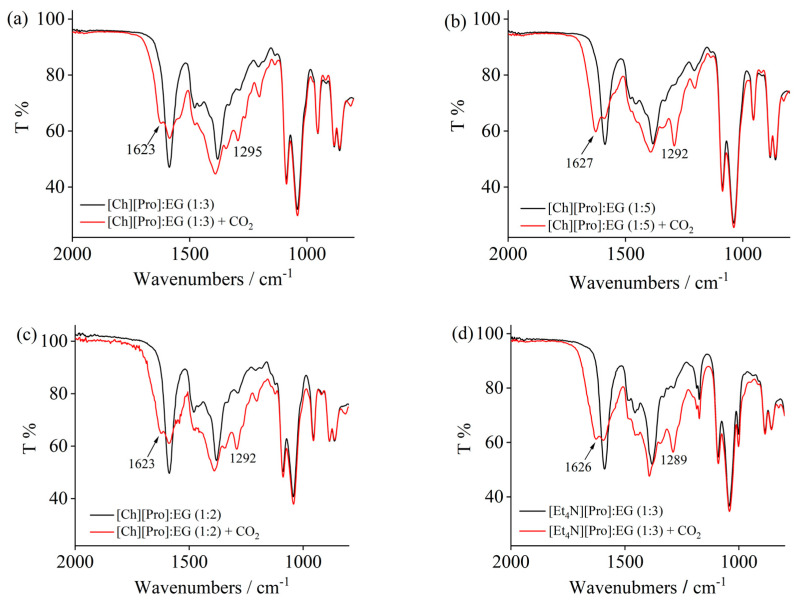
FTIR spectra of [Ch][Pro]:EG (1:3) (**a**), [Ch][Pro]:EG (1:5) (**b**), [Ch][Pro]:EG (1:2) (**c**), and [Et_4_N][Pro]:EG (1:3) (**d**) before and after CO_2_ absorption.

The FTIR spectra of the DESs used before and after CO_2_ capture are shown in Figure 4. As seen in Figure 4a, two new bands at 1623 and 1295 cm^−1^ can be found after absorption for [Ch][Pro]:EG (1:3) system. The band at 1623 cm^−1^ is the combined peak of C=O stretching from the carbamate acid and the carbonate species [40,51,52,53]. The band at 1295 cm^−1^ can be attributed to the stretching of the O-COO^−^ bond [39,40]. Similarly, for the [Ch][Pro]:EG (1:5) system, the new peaks appear at 1627 and 1292 cm^−1^ after CO_2_ capture (Figure 4b). Similar peaks can also be observed at 1623 and 1292 cm^−1^ for the solvent [Ch][Pro]:EG (1:2) after capture (Figure 4c). Moreover, two new bands at 1626 and 1289 cm^−1^ can be found as well in the FTIR spectra of [Et_4_N][Pro]:EG (1:3) after CO_2_ absorption (Figure 4d). Therefore, the FTIR results also provide evidence that CO_2_ reacts both with the anion [Pro]^−^ and EG. The full-window FTIR spectra of the solvents before and after CO_2_ capture are shown in Figures S11–S15.

**Scheme 2:**
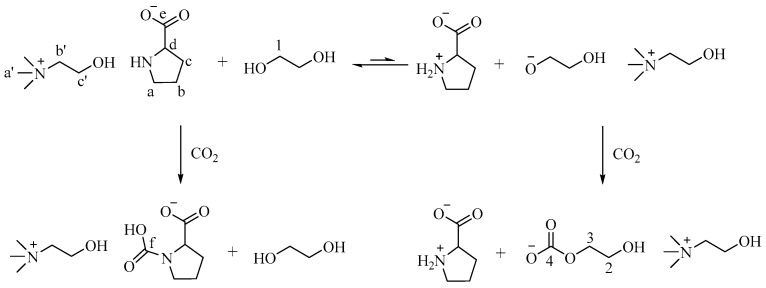
The possible CO_2_ capture mechanism by [Ch][Pro]-EG used in this work.

On the basis of the above findings, the possible reaction mechanism between CO_2_ and [Ch][Pro]-EG studied is presented in Scheme 2. In the [Ch][Pro]-EG solvent, there is an acid-based reaction between anion [Pro]^−^ and EG, producing the anion HO–CH_2_–CH_2_–O^−^. The amino group of anion [Pro]^−^ may influence the acidity of EG through the interaction between the N atom of an amino group and the H atom of the -OH group, so EG can be deprotonated.

When CO_2_ is absorbed by [Ch][Pro]-EG solvent, CO_2_ can react with [Pro]^−^ to form a carbamate acid, and it can also react with HO–CH_2_–CH_2_–O^−^ to form carbonate species.

## 3. Materials and Methods

### 3.1. Materials and Characterizations

Proline (99%) and ethylene glycol (99.5%) were obtained from J&K Scientific Ltd. (Beijing, China) Choline hydroxide ([Ch][OH], 44% *w*/*w* in water) and tetramethylammonium hydroxide ([Et_4_N][OH]) (25% *w*/*w* in water) were purchased from Innochem Science and Technology Co., Ltd. (Beijing, China). CO_2_ (≥99.99%) was provided by Beijing ZG Special Gases Sci. and Tech. Co. Ltd. (Beijing, China).

The ^13^C NMR spectra were taken on a Bruker spectrometer (151 MHz) using d_6_-DMSO as the internal reference. The FTIR spectra were recorded on a Perkin-Elmer frontier spectrometer equipped with an attenuated total reflection (ATR) accessory. Ethylene glycol was dried with 4 Å molecular sieve prior to use. The exact concentrations of [Ch][OH] and [Et_4_N][OH] in aqueous solution were titrated with potassium hydrogen phthalate (KHP). The water content in the solvents was determined by Karl–Fischer titration method.

### 3.2. Synthesis of Proline-Based ILs

For the synthesis of [Ch][Pro], equimolar proline (Pro) was added to the aqueous solution of [Ch][OH] ([Ch][OH]:Pro = 1:1). The mixture was stirred at room temperature for 2 h, and then water in the solution was removed by using rotary evaporation at 70 °C to obtain the IL [Ch][Pro]. Finally, the IL [Ch][Pro] was dried under vacuum at 70 °C prior to use.

The synthesis of [Et_4_N][Pro] was similar to that of [Ch][Pro].

### 3.3. Synthesis of DESs

For the DESs [Ch][Pro]-EG, the IL [Cho][Pro] and ethylene glycol were mixed at desired molar ratios, and then the mixtures were stirred at 70 °C until homogenous liquids were formed, and then liquid solvents were cooled down to room temperature to obtain the DESs. The water contents of the DESs [Ch][Pro]:EG (1:2), (1:3), (1:4), and (1:5) were 0.21, 0.18, 0.17, and 0.17 wt%, respectively.

The synthesis of the solvent [Et_4_N][Pro]:EG (1:3) was similar to those of [Ch][Pro]-EG DESs.

NMR and FTIR data of solvents used in this work:

[Ch][Pro]:EG (1:2): ^13^C NMR (151 MHz, *d*_6_-DMSO) δ = 178.2, 67.4, 63.2, 62.4, 55.4, 53.4, 47.0, 31.3, 26.1 ppm. FTIR: ν = 3282, 2920, 2866, 1587, 1480, 1378, 1089, 1037, 958, 885, 863, 635 cm^−1^.
[Ch][Pro]:EG (1:3): ^13^C NMR (151 MHz, *d*_6_-DMSO) δ = 177.8, 67.3, 63.2, 62.2, 55.4, 53.4, 46.8, 31.1, 26.0 ppm. FTIR: ν = 3283, 2924, 2872, 1587, 1480, 1381, 1086, 1039, 954, 884, 861, 627 cm^−1^.
[Ch][Pro]:EG (1:4): ^13^C NMR (151 MHz, *d*_6_-DMSO) δ = 178.4, 67.5, 63.2, 62.4, 55.5, 53.5, 46.9, 31.3, 26.1 ppm. FTIR: ν = 3285, 2928, 2870, 1587, 1480, 1382, 1087, 1039, 953, 883, 861, 624 cm^−1^.
[Ch][Pro]:EG (1:5): ^13^C NMR (151 MHz, *d*_6_-DMSO) δ = 177.9, 67.3, 63.1, 62.2, 55.4, 53.4, 46.8, 31.3, 26.0 ppm. FTIR: ν = 3290, 2932, 2864, 1587, 1480, 1382, 1087, 1038, 954, 884, 861, 623 cm^−1^.
[Et_4_N][Pro]:EG (1:3): ^13^C NMR (151 MHz, *d*_6_-DMSO) δ = 178.1, 63.2, 62.4, 51.6, 47.1, 31.3, 26.1, 7.1 ppm. FTIR: ν = 3285, 2922, 2869, 1588, 1457, 1382, 1175, 1092, 1043, 1002, 884, 855, 784, 636 cm^−1^.

### 3.4. Absorption of CO_2_

DESs (~2 g) were added into a glass tube with an inner diameter of 10 mm. CO_2_ (~50 mL/min) was bubbled into the glass tube, which was partially immersed in a water bath at the required temperature. The amount of CO_2_ absorbed by solvents can be calculated by the weights of the tube before and after CO_2_ absorption, which were determined by an electronic balance with an accuracy of ±0.1 mg. The weight of the tube was measured at regular intervals during the absorption process. If the weight of the tube did not change with time, the CO_2_ absorption by DESs was considered to have reached saturation.

## 4. Conclusions

The CO_2_ capture mechanism by DESs consisting of [Ch][Pro] and EG is studied by using NMR and FTIR methods. The NMR and FTIR results reveal that CO_2_ can react with both the anion [Pro]^−^ and EG. In the solvents, EG can be deprotonated by the amino group of the anion [Pro]^−^, resulting in the formation of the anion HO–CH_2_—CH_2_–O^−^. During the absorption process, CO_2_ can react both with the amino group of the anion [Pro]^−^ and the O^−^ of the anion HO–CH_2_–CH_2_–O^−^, forming carbamate acid and carbonate species, respectively. For the [Et_4_N][Pro]:EG (1:3) solvent, CO_2_ can also react with both the anion [Pro]^−^ and EG. We think the findings of this work will be useful for understanding the interactions between CO_2_ and the components of DESs.

## Data Availability

The data are available on request.

## Supplementary Materials

The following supporting information can be downloaded at: https://www.mdpi.com/article/10.3390/molecules28145461/s1, Figure S1: The ^13^C NMR spectra of [Ch][Pro]:EG (1:3) before (a) and after (b) CO_2_ capture.; Figure S2: The ^13^C NMR spectra of [Ch][Pro]:EG (1:4) before (a) and after (b) CO_2_ capture.; Figure S3: The ^13^C NMR spectra of [Ch][Pro]:EG (1:5) before (a) and after (b) CO_2_ capture.; Figure S4: The ^13^C NMR spectra of [Ch][Pro]:EG (1:2) before (a) and after (b) CO_2_ capture.; Figure S5: The ^13^C NMR spectra of [Et_4_N][Pro]:EG (1:3) before (a) and after (b) CO_2_ capture.; Figure S6: The ^1^H NMR spectra of [Ch][Pro]:EG (1:3) before (a) and after (b) CO_2_ capture.; Figure S7: The ^1^H NMR spectra of [Ch][Pro]:EG (1:4) before (a) and after (b) CO_2_ capture.; Figure S8: The ^1^H NMR spectra of [Ch][Pro]:EG (1:5) before (a) and after (b) CO_2_ capture.; Figure S9: The ^1^H NMR spectra of [Ch][Pro]:EG (1:2) before (a) and after (b) CO_2_ capture.; Figure S10: The ^1^H NMR spectra of [Et_4_N][Pro]:EG (1:3) before (a) and after (b) CO_2_ capture.; Figure S11: The FTIR spectra of [Ch][Pro]:EG (1:3) before and after CO_2_ capture.; Figure S12: The FTIR spectra of [Ch][Pro]:EG (1:4) before and after CO_2_ capture.; Figure S13: The FTIR spectra of [Ch][Pro]:EG (1:5) before and after CO_2_ capture.; Figure S14: The FTIR spectra of [Ch][Pro]:EG (1:2) before and after CO_2_ capture.; Figure S15: The FTIR spectra of [Et_4_N][Pro]:EG (1:3) before and after CO_2_ capture.
