# Supplementary material for: CO2 Capture Mechanism by Deep Eutectic Solvents Formed by Choline Prolinate and Ethylene Glycol
Source: Molecules. 2023 Jul 17;28(14):5461. doi: 10.3390/molecules28145461 (PMC10385772; doi:10.3390/molecules28145461)
Supplement: Supplementary file 1 [file molecules-28-05461-s001.zip › molecules-2468373-SI.pdf]

Supporting information

CO_2 Capture Mechanism by Deep Eutectic Solvents Formed by Choline Prolinate and Ethylene Glycol

Mingzhe Chen and Jinming Xu *

School of Science, China University of Geosciences, Beijing 100083, China; 2019220039@email.cugb.edu.cn
* Correspondence: xujm@cugb.edu.cn

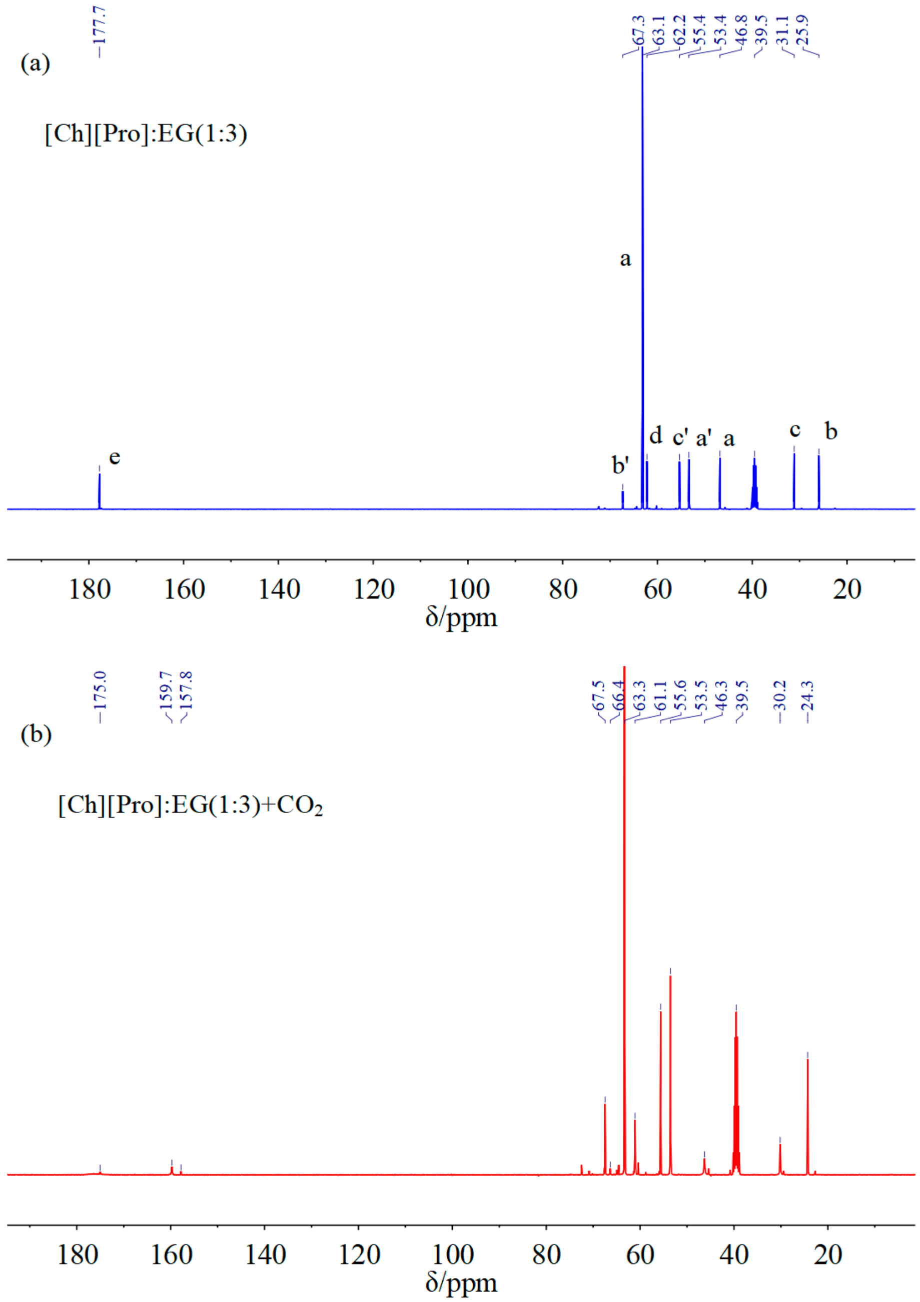

Figures S1. The ^{13}C NMR spectra of [Ch][Pro]:EG (1:3) before (a) and after (b) CO_2 capture.

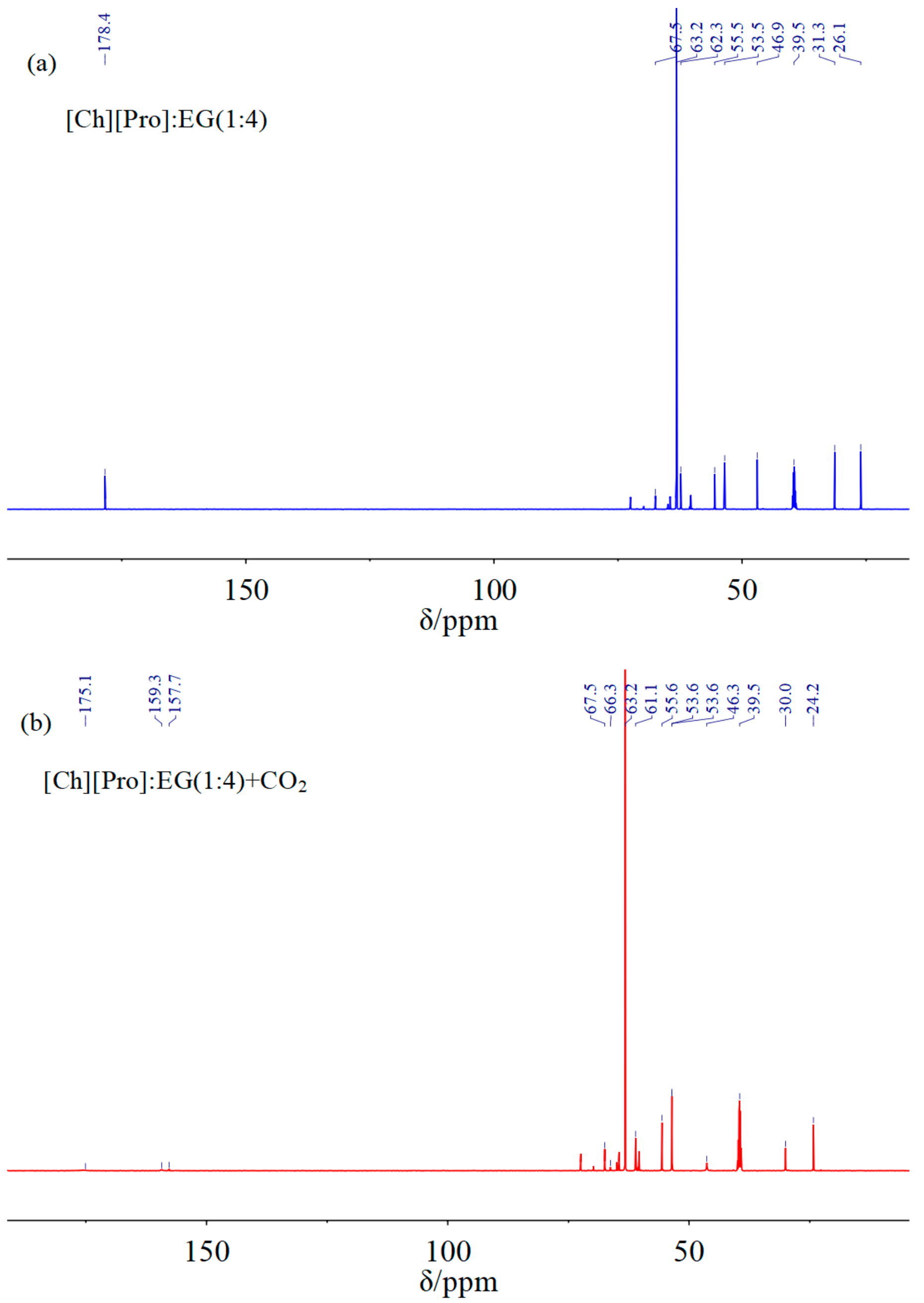

Figures S2. The ^{13}C NMR spectra of [Ch][Pro]:EG (1:4) before (a) and after (b) CO_2 capture.

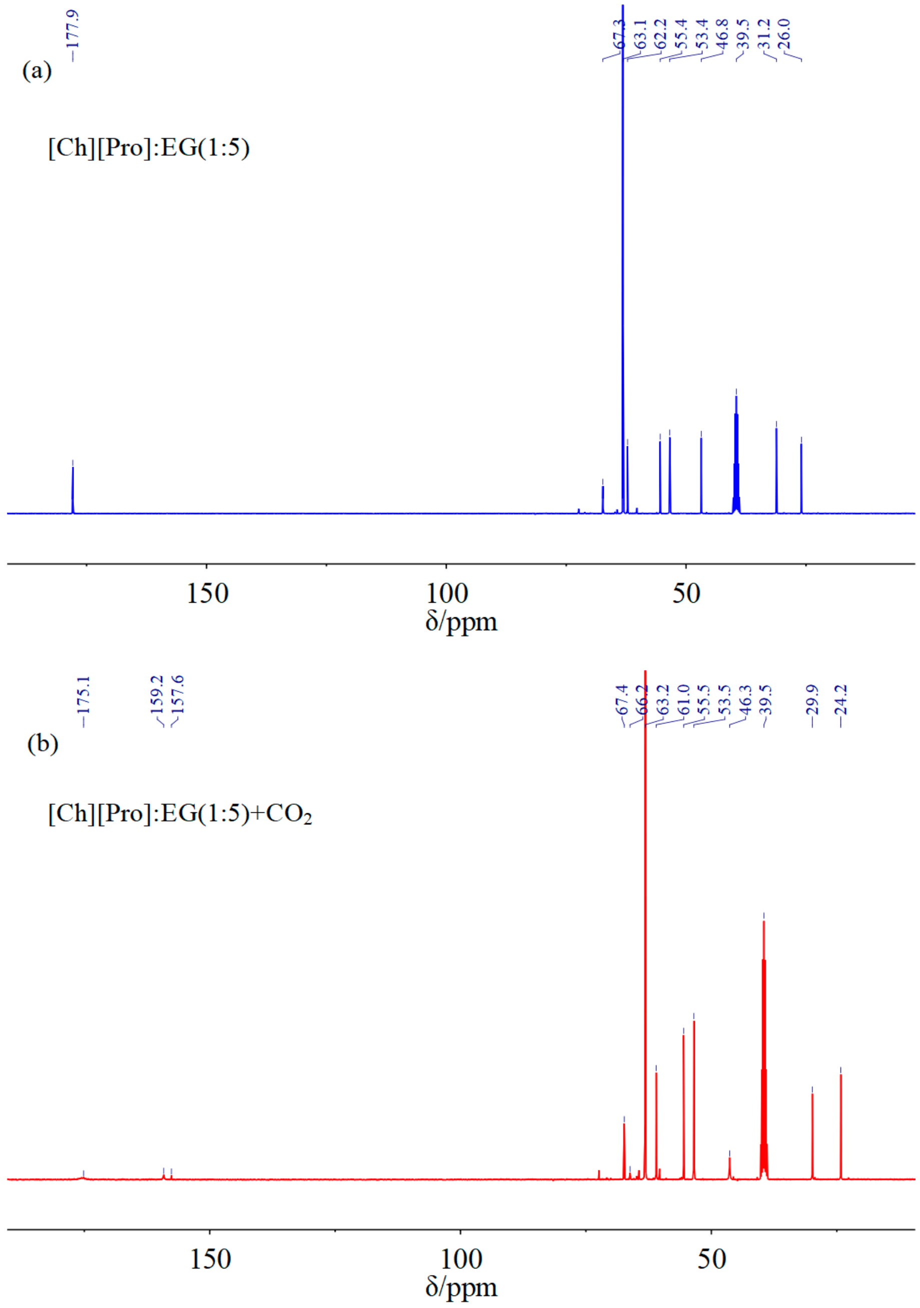

Figures S3. The ^{13}C NMR spectra of [Ch][Pro]:EG (1:5) before (a) and after (b) CO_2 capture.

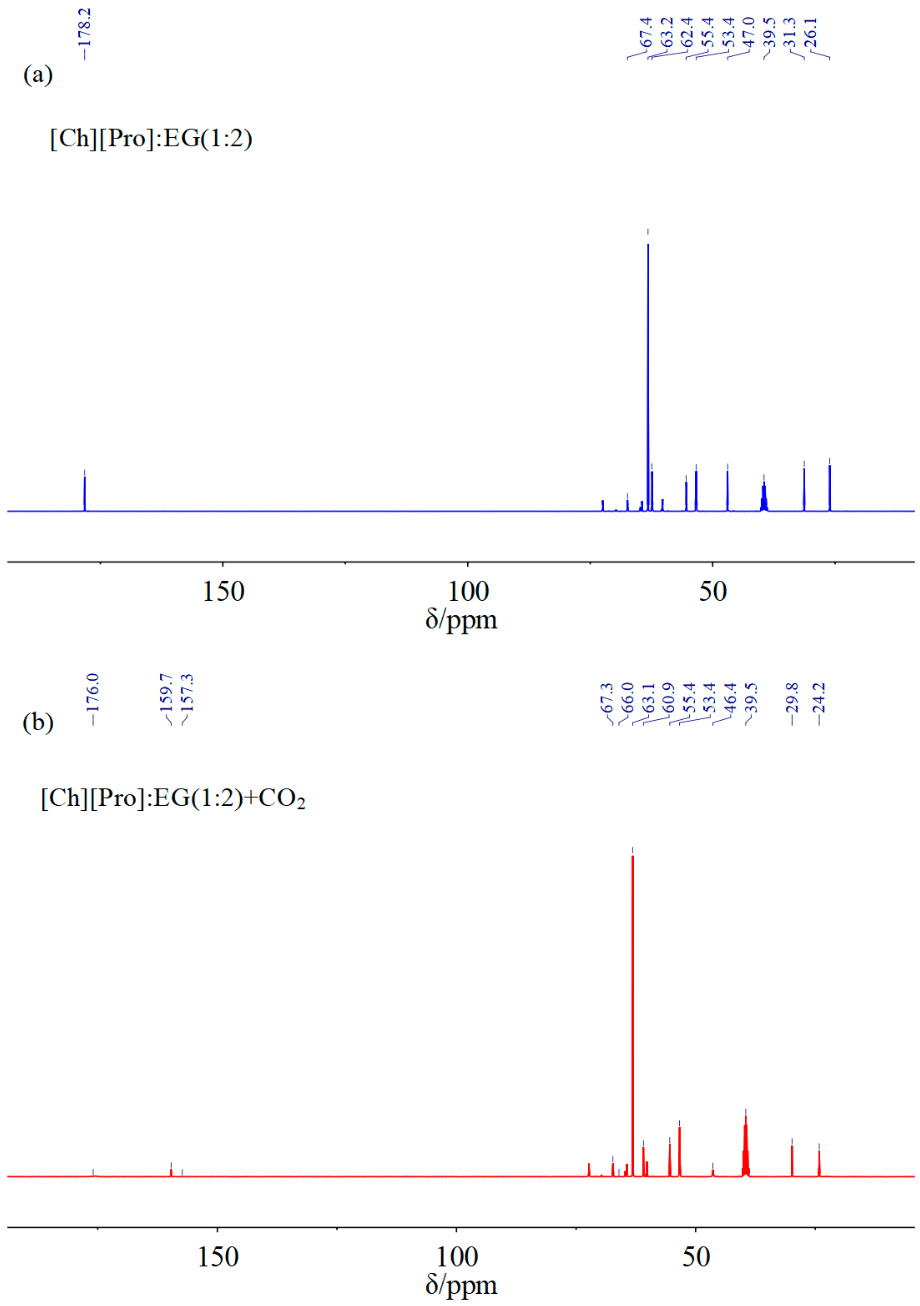

Figures S4. The ^{13}C NMR spectra of [Ch][Pro]:EG (1:2) before (a) and after (b) CO_2 capture.

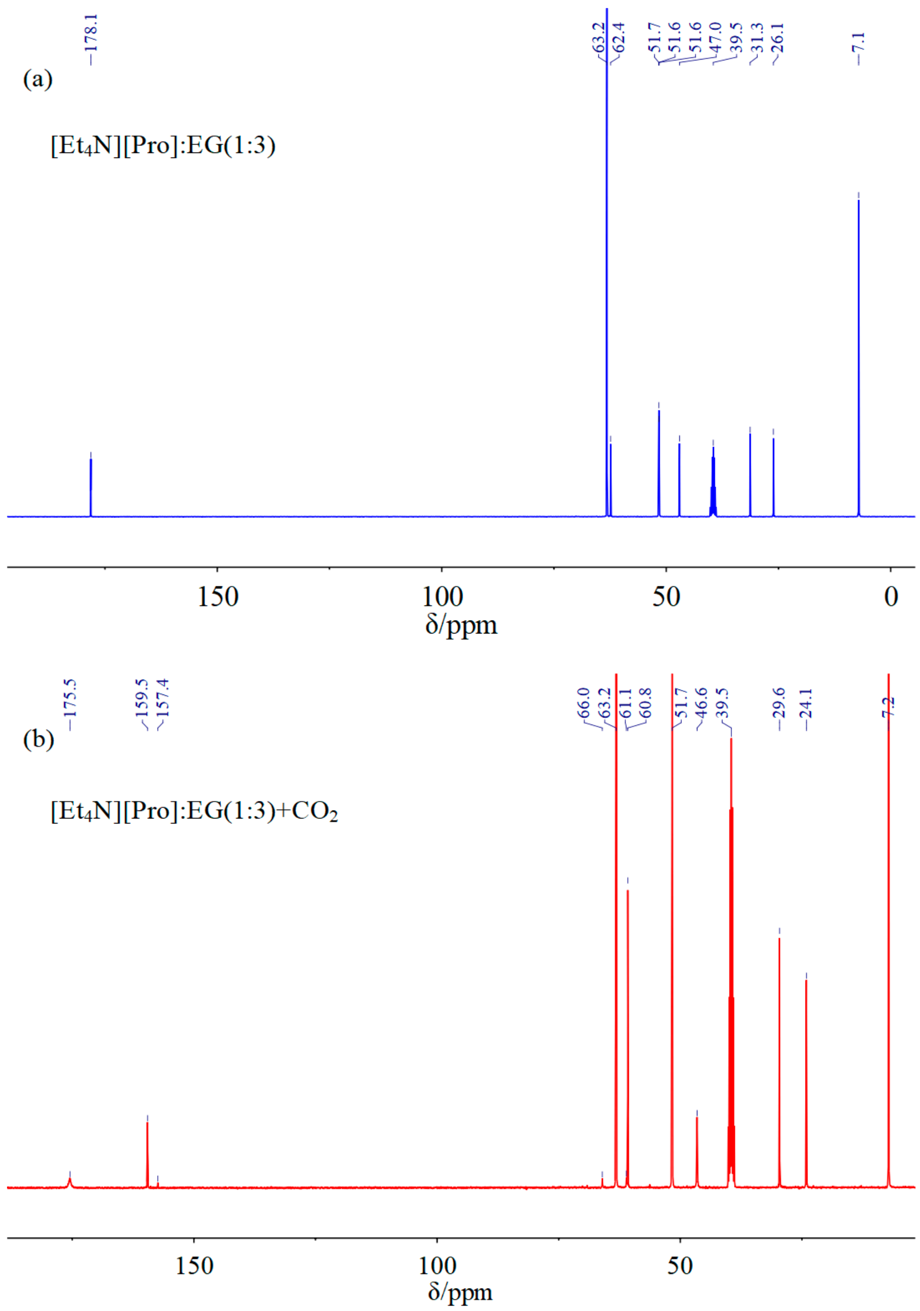

Figures S5. The ^{13}C NMR spectra of [Et4N][Pro]:EG (1:3) before (a) and after (b) CO_2 capture.

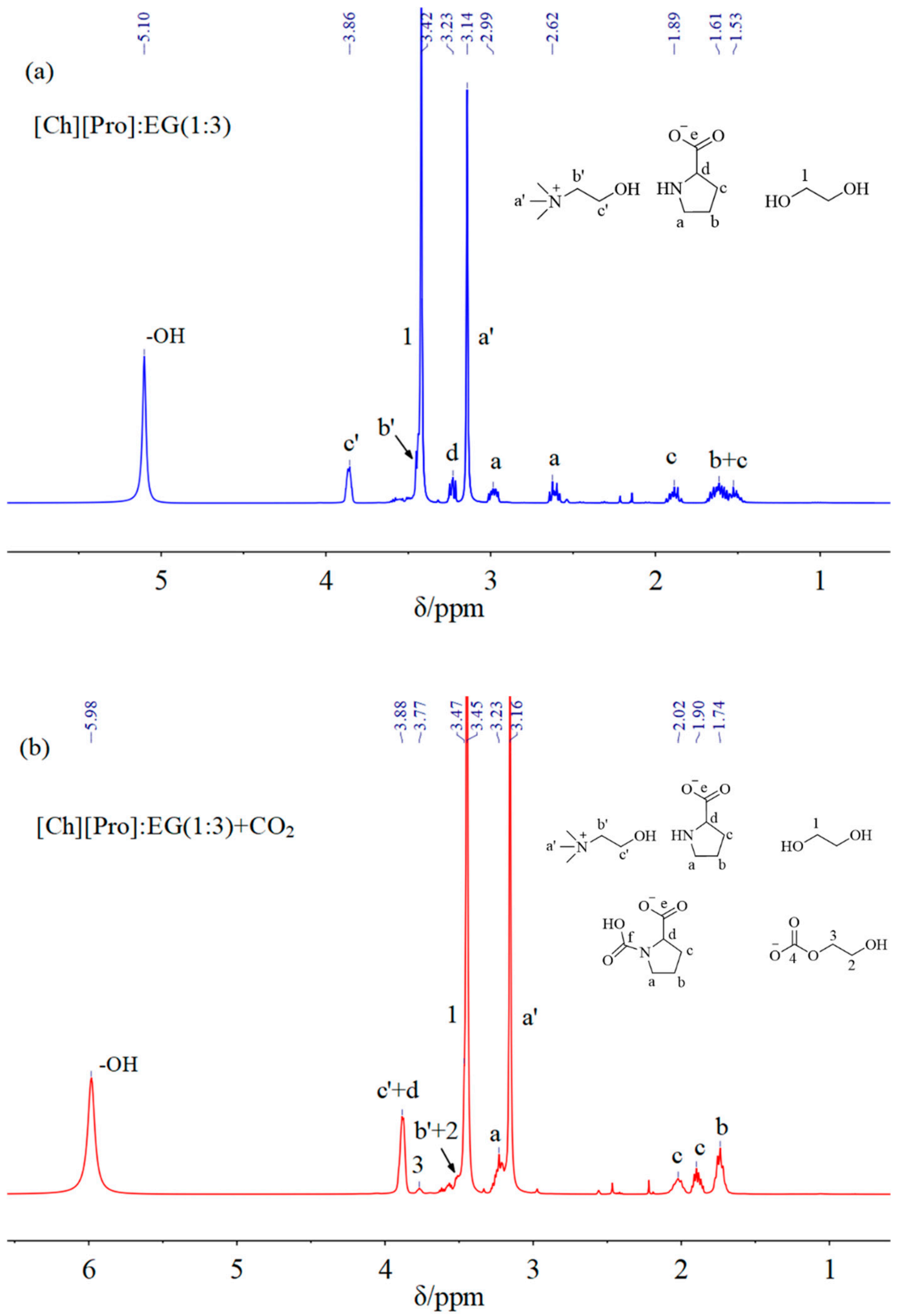

Figures S6. The ^{1}H NMR spectra of [Ch][Pro]:EG (1:3) before (a) and after (b) CO_2 capture.

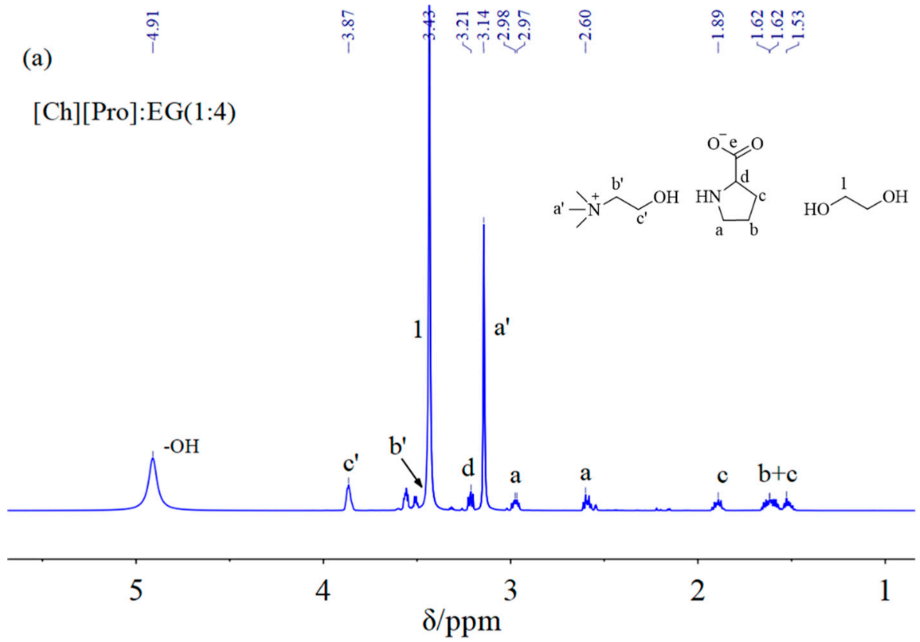

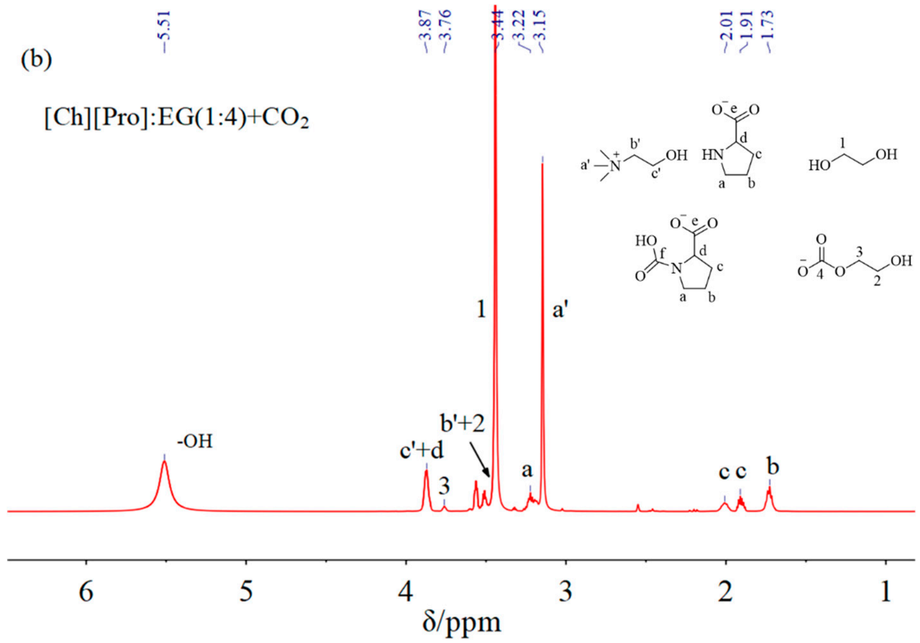

Figures S7. The ^{1}H NMR spectra of [Ch][Pro]:EG (1:4) before (a) and after (b) CO_2 capture.

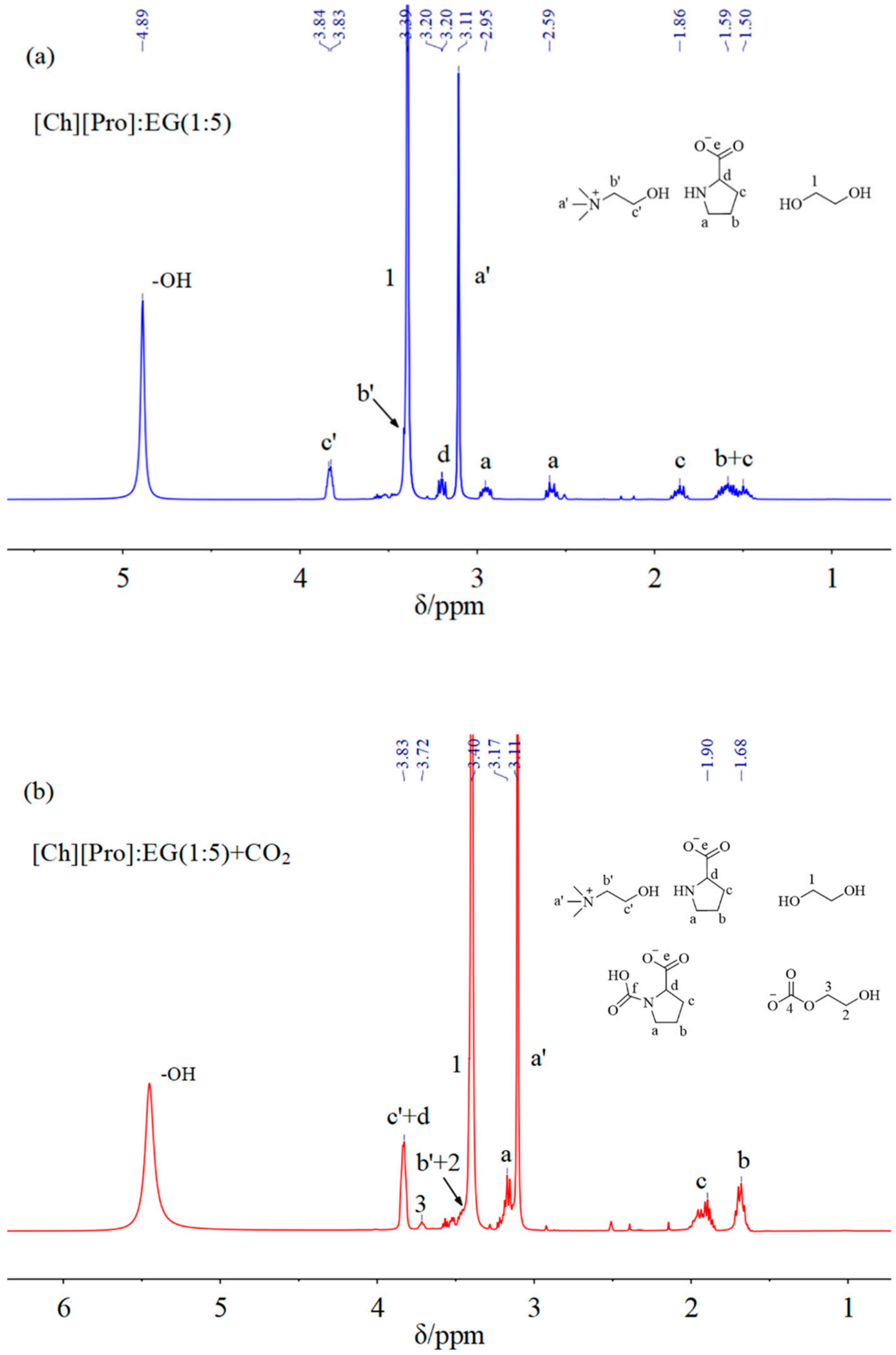

Figures S8. The ^{1}H NMR spectra of [Ch][Pro]:EG (1:5) before (a) and after (b) CO_2 capture.

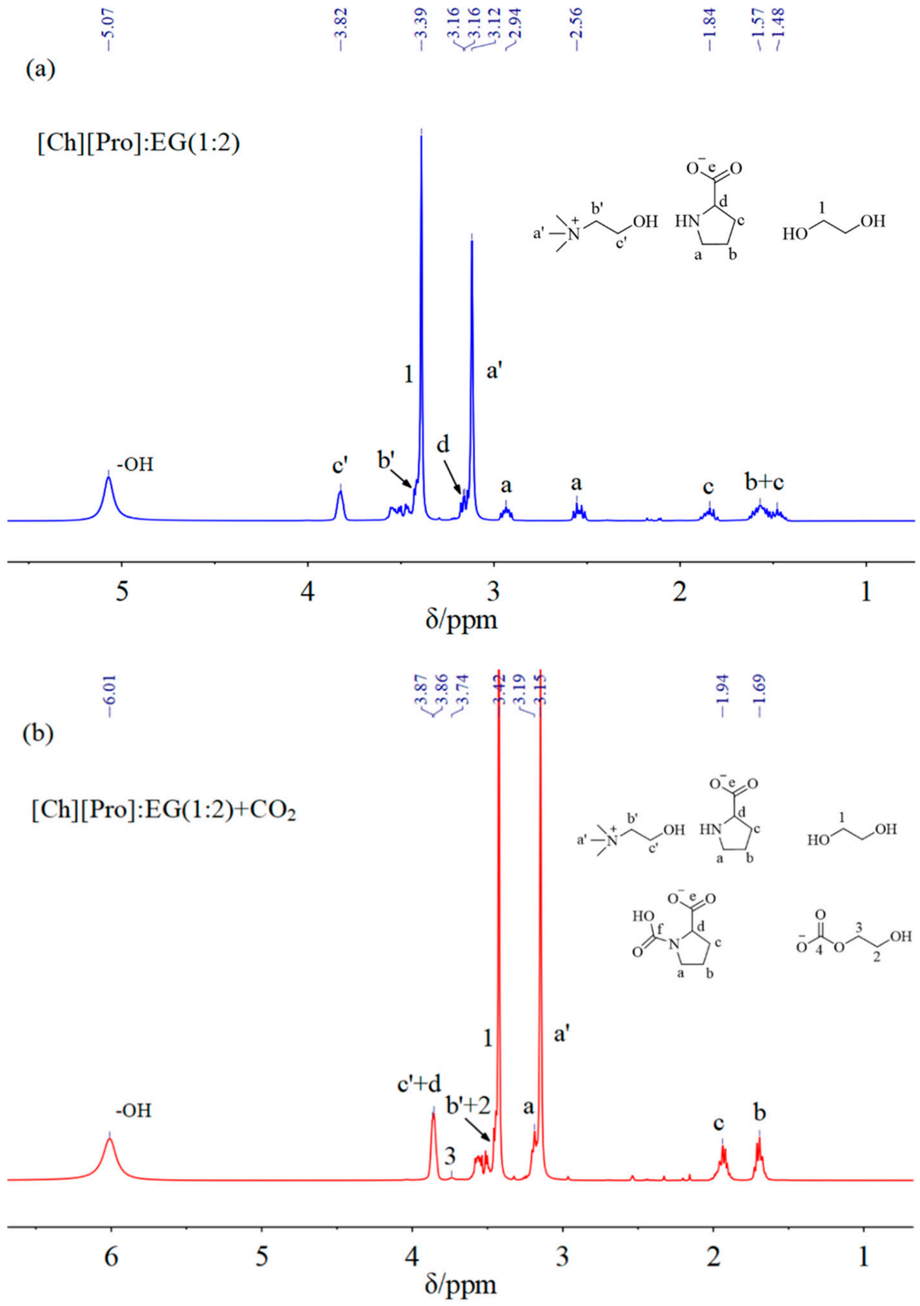

Figures S9. The ^{1}H NMR spectra of [Ch][Pro]:EG (1:2) before (a) and after (b) CO_2 capture.

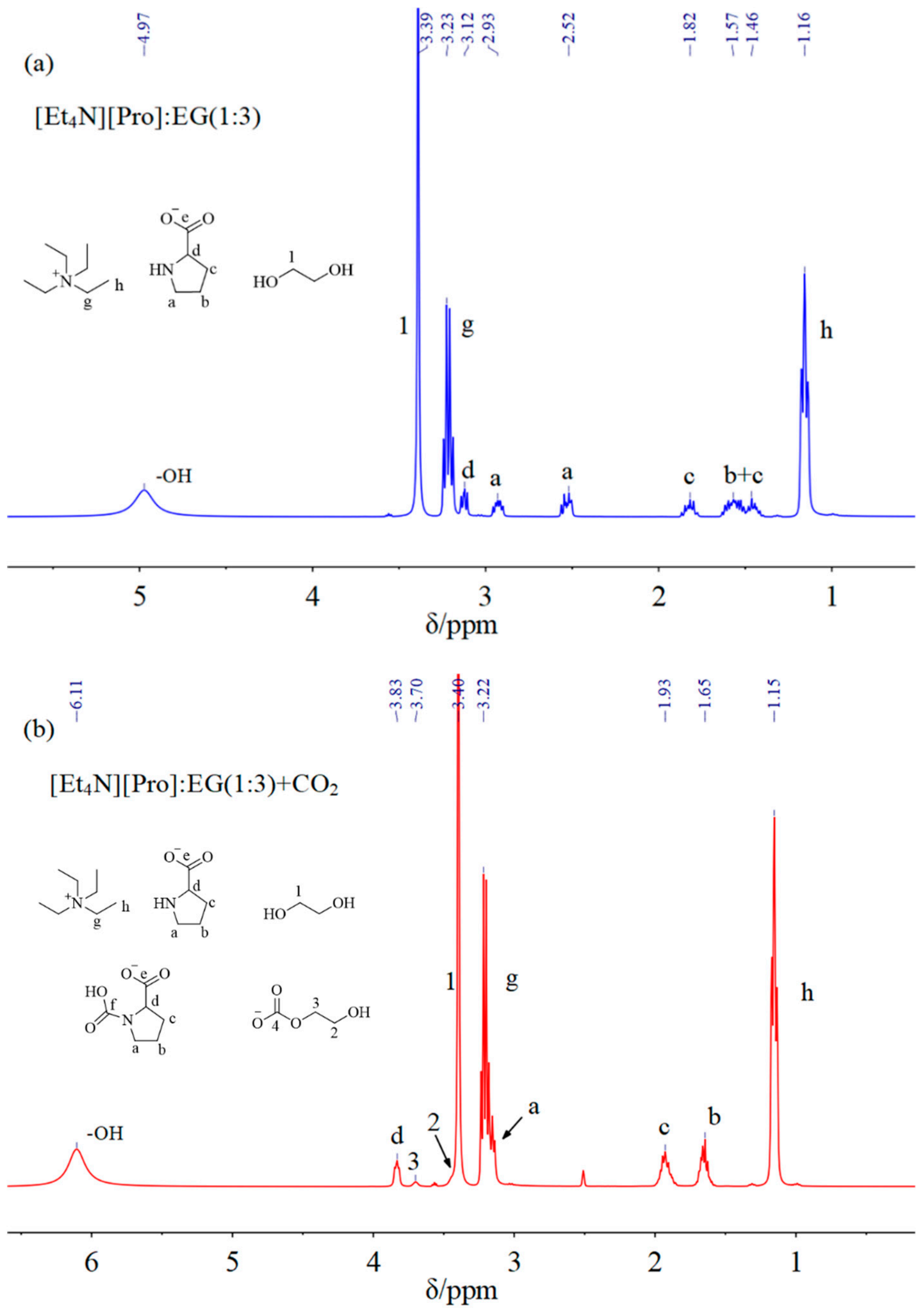

Figures S10. The ^{1}H NMR spectra of $[Et_4N][Pro]$:EG (1:3) before (a) and after (b) CO_2 capture.

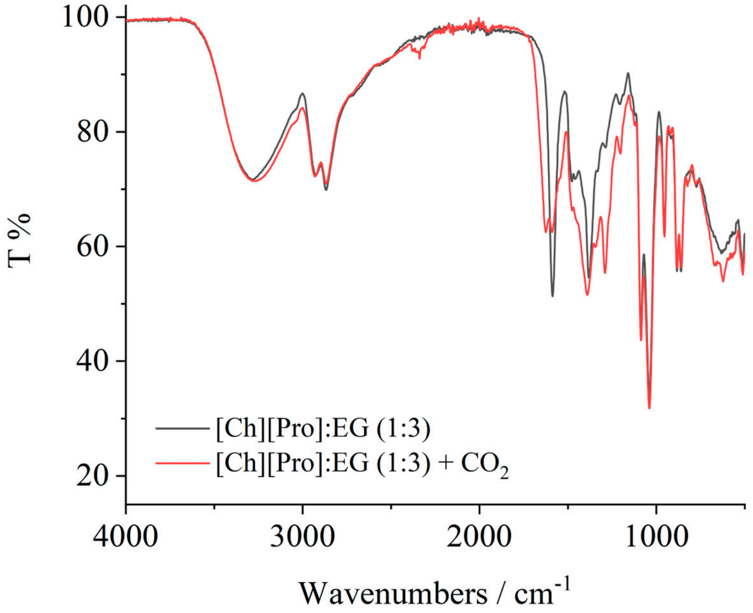

Figures S11. The FTIR spectra of [Ch][Pro]:EG (1:3) before and after CO_2 capture.

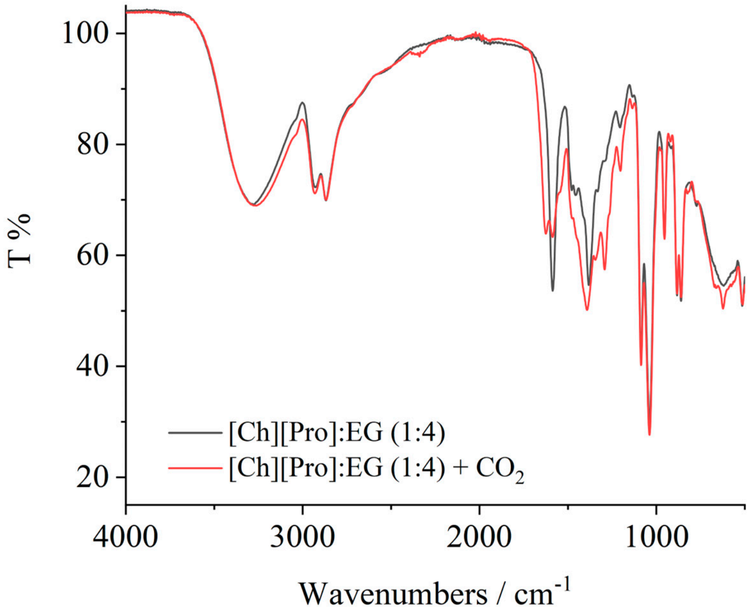

Figures S12. The FTIR spectra of [Ch][Pro]:EG (1:4) before and after CO_2 capture.

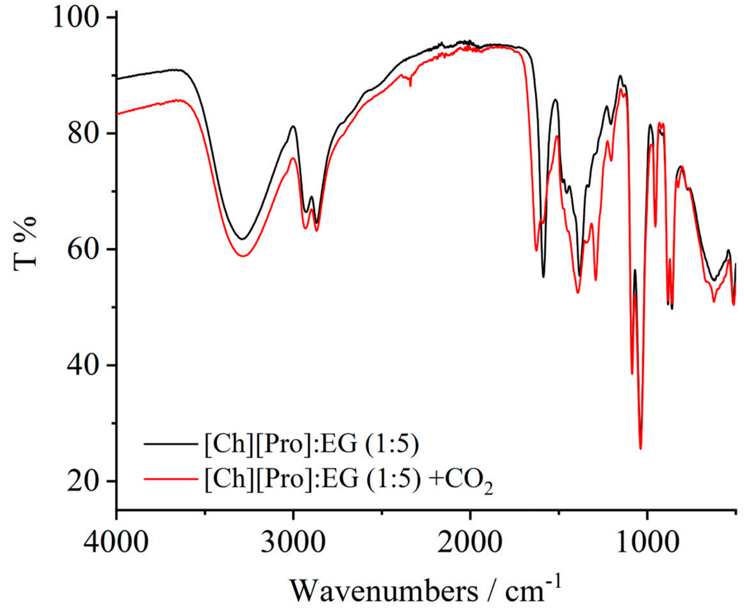

Figures S13. The FTIR spectra of [Ch][Pro]:EG (1:5) before and after CO_2 capture.

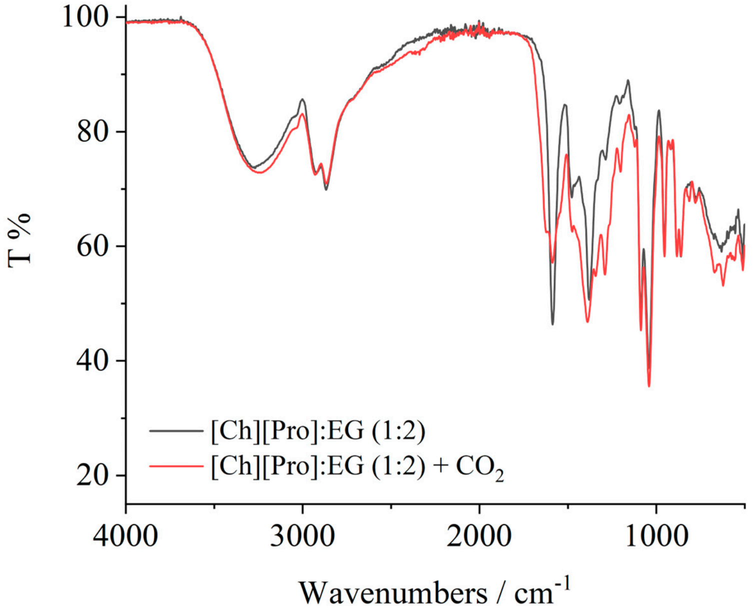

Figures S14. The FTIR spectra of [Ch][Pro]:EG (1:2) before and after CO_2 capture.

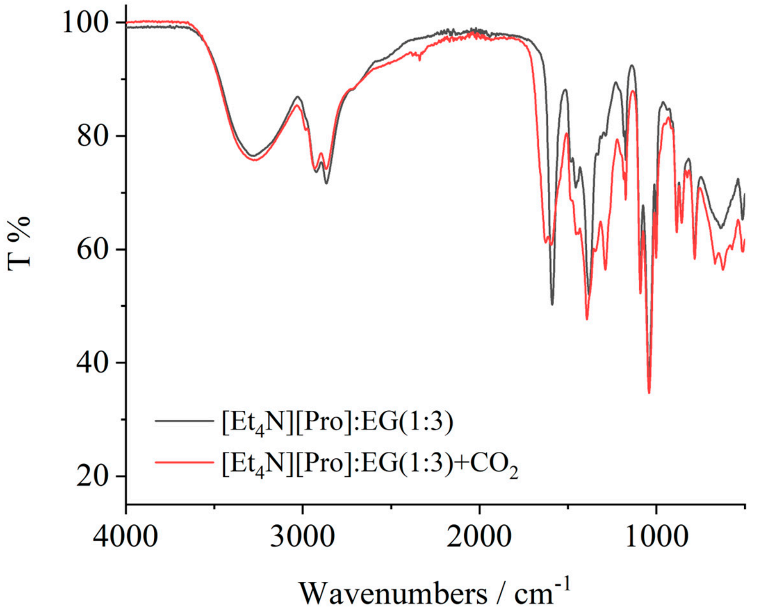

Figures S15. The FTIR spectra of [Et_4N][Pro]:EG (1:3) before and after CO_2 capture.
